# Prevalence and risk factors for age-related cataract in Sweden

**DOI:** 10.48101/ujms.v126.8349

**Published:** 2021-12-31

**Authors:** Amee Patel, Krish Patel

**Affiliations:** The Royal Marsden Hospital, London, United Kingdom; The Royal London Hospital, Barts Health NHS Trust, London, United Kingdom

Dear Editor,

We read with great interest the article by Hugosson and Ekström on ‘Prevalence and risk factors for age-related cataract in Sweden’ (1). The study concluded that increased age, being female and myopia are significant risk factors for developing cataract in this population group.

The authors used retrospective analysis to determine cataract prevalence using the Tierp Glaucoma Survey, obtaining data of people aged 65–74 years between 1984 and 1986. This survey was originally used to measure the prevalence of open-angle glaucoma in Tierp, and methods included a thorough ophthalmic assessment by a single ophthalmologist. Our concern is that since the primary outcome of the original study was to investigate prevalence of a different eye disorder, there may be an element of reporting bias; in some participants, cataracts may be undisclosed due to irrelevance.

Importantly, the authors recognise the lack of standardising cataract diagnostics between participants. This may contribute to an underestimation of cataract prevalence. It is not pertinent to extrapolate this data with confidence to a wider population throughout Sweden due to the small sample size and a limited age range assessed.

It is not clear how long the named risk factors have preceded cataract formation, if indeed they had. Furthermore, it would be intriguing to know whether being more myopic is a greater risk factor compared to lower refractive powers. Again, it is difficult to ascertain whether myopia was a consequence of cataract, rather than a risk factor, which necessitates more clarity in the analysis of participant demographics.

This article dismisses hypertension as a significant risk factor for cataract; however, there are numerous studies making this association such as an American study by Ang and Afshari (2). They note increased risk of cataract formation with increased severity of hypertension. Ethnicity and ultraviolet exposure are frequently cited in literature as risk factors; however, these were not mentioned in the study by Hugosson and Ekström. Readers are curious to know whether these are important risk factors in this Swedish population.

We echo the importance of studies such as this, considering that world-wide cataract remains one of the most common elective surgical procedures and a leading cause of visual impairment. Understanding these risk factors guides local health services to devise targeted preventive strategies, ultimately aiming to reduce the impact on patients. This is significant in the coronavirus disease 2019 (COVID-19) era, where health services have been disrupted globally with elective procedures abandoned, patients have had to learn to live with poor vision for longer. Identifying and optimising risk factors emphasise that preventative medicine is crucial in the future of ophthalmology.


Amee Patel The Royal Marsden Hospital, London, United Kingdom
Krish Patel The Royal London Hospital, Barts Health NHS Trust, London, United Kingdom

